# Approaches to Multimodality Monitoring in Pediatric Traumatic Brain Injury

**DOI:** 10.3389/fneur.2019.01261

**Published:** 2019-11-26

**Authors:** Brian Appavu, Brian T. Burrows, Stephen Foldes, P. David Adelson

**Affiliations:** ^1^Barrow Neurological Institute, Phoenix Children's Hospital, Phoenix, AZ, United States; ^2^Department of Child Health, University of Arizona College of Medicine – Phoenix, Phoenix, AZ, United States

**Keywords:** multimodality monitoring, autoregulation, traumatic brain injury, pediatrics, neurocritical care

## Abstract

Traumatic brain injury (TBI) is a leading cause of morbidity and mortality in children. Improved methods of monitoring real-time cerebral physiology are needed to better understand when secondary brain injury develops and what treatment strategies may alleviate or prevent such injury. In this review, we discuss emerging technologies that exist to better understand intracranial pressure (ICP), cerebral blood flow, metabolism, oxygenation and electrical activity. We also discuss approaches to integrating these data as part of a multimodality monitoring strategy to improve patient care.

## Introduction

Traumatic brain injury (TBI) represents an alteration of brain function or other evidence of brain pathology that is caused by an external force (1). In children, TBI is a leading cause of morbidity and mortality in the United States and poses significant financial burden to the United States healthcare system (2). While treatment against primary TBI insult is prevention, subsequent insults may occur over ensuing days leading to secondary brain injury. Fundamentally, neurocritical care is aimed at rapidly detecting such insults and treating against them.

Much of secondary brain injury develops when mismatch exists between cerebral metabolic demand and energy substrate delivery as well as other pathophysiologic cascades of mechanisms leading to cell damage and death. Emerging technologies have developed to better monitor cerebral physiology in real-time. These technologies capture continuous recordings of intracranial pressure (ICP), cerebral oxygenation, cerebral blood flow, cerebral metabolism, and electrical activity. Each of these modalities alone can provide insight into potentially pathological physiology, but when interrelationships between these modalities are observed, they can offer greater insights into cerebral physiologic dynamics.

Monitoring CBF and metabolism in neurocritical care requires combining techniques that provide enough spatial and temporal resolution data. When a patient presents with TBI, the initial assessment requires high spatial resolution imaging to understand affected neuroanatomy. Anatomical imaging should continue to be employed judiciously to avoid secondary insults (3). Once obtained and initial interventions are performed, techniques with high temporal resolution can be employed for long-term continuous monitoring to understand ensuing physiology.

In this review, we describe existing neuromonitoring modalities that characterize the physiologic profile of TBI (Table 1). While neuroimaging is important for neurocritical care management, it is beyond the scope of this review. We describe approaches that may be taken to use neuromonitoring modalities to create an environment of recovery and amelioration against secondary injury. We also describe challenges that exist when addressing these strategies in the pediatric population.

**Table 1:** Frequent multimodal monitoring techniques.

**Technique**	**Physiology**	**Units**	**Resolution**	**Advantages**	**Disadvantages**	**Pediatric considerations**
External ventricular catheter (EVD)	Intracranial pressure (ICP)	Mm Hg	Low-moderate spatial, high temporal	Allows global measurements of ICP, allows therapeutic/diagnostic drainage.	Increased infection risk, difficult placement in effaced ventricles. Cannot measure ICP unless clamped.	ICP thresholds may vary with age.
Intraparenchymal ICP Monitor	Intracranial Pressure	Mm Hg	Low spatial, high temporal	Continuous measurements. Easy placement. Lower infection risk.	Units may drift over time. No direct therapeutic benefit.	ICP thresholds may vary with age.
Near Infrared Spectroscopy (NIRS)	Oxygen saturation	%	Low spatial, high temporal	Allows non-invasive measurements of brain parenchymal oxygenation.	Difficult to interpret in setting of hematoma/ edema.	Thresholds for normative values are not well established
Brain tissue Oxygention (PbtO_2_)	Oxygen tension	Torr	Low spatial, high temporal	Direct measurements of brain oxygen content	Invasive, may reflect regional changes rather than global.	Needs to be placed using bolt, which may not be feasible with thin skull.
Transcranial Doppler Ultrasound (TCD)	Mean flow velocities (MVF)	Cm/sec	Low-moderate spatial, moderate-high temporal	Non-invasive, can provide bedside assessments of vasospasms, hyperemia, autoregulation, or arterial occlusions	Limited diagnostic specificity in absence of anatomical imaging	Mean flow velocities change with age
Laser Doppler flowmetry (LD)	Mean flow velocities (MFV)	Cm/sec	Low spatial, high temporal	Can assess microcirculatory blood flow changes	Invasive, prone to probe migration	Not well-described in children
Thermal diffusion flowmetry (TD)	Cerebral blood flow	mL / 100 g / min	Low spatial, high temporal	Provides continuous, direct measurements of parenchymal perfusion	Invasive, recording suspends with increased pulsatility or temperature	Not well-described in children
Cerebral microdialysis (CMD)	Concentrations of cerebral metabolites	Mmol/L	Low spatial, moderate temporal	Provides direct biomarkers of metabolic crisis	Invasive, requires hourly vial retrieval for analysis	Normative values not well-established
Continuous Electroencephalography (cEEG)	Discrete and continuous variables (e.g., seizures, alpha power/variability, etc.)	N/A	Low-moderate spatial, high temporal	Diagnostic for seizures. Sensitive for encephalopathy, ischemia, and sedation monitoring.	Lacks neuroanatomical visualization. Requires expert proficiency for interpretation	Background activity changes with age
Pupillometry	Pupil size, pupil reactivity	Mm, neurological pupil index (NPI)	Low spatial, moderate temporal	Objective measurements of pupil size. Can relate to injury burden in setting of ICP crisis	Lacks diagnostic specificity of ABI in absence of other neuromonitoring modalities	Normative values are still limited

## Neuromonitoring Techniques

### Intracranial Pressure

Intracranial pressure (ICP) represents the pressure within the intracranial vault. Pathological increases in ICP can have profound consequences, including insufficient cerebral perfusion pressures (CPP) leading to ischemia and herniation. Maintaining appropriate ICP and CPP parameters is a critical therapeutic strategy for secondary injury prevention. The external ventricular drain (EVD) represents the gold standard technique for ICP monitoring. The advantage of the EVD is that it allows global ICP measurements while allowing for therapeutic drainage of cerebral spinal fluid (CSF). Disadvantages include its invasive nature, challenge of implementation when ventricular effacement exists, and inability to produce continuous ICP measurements without synchronized drainage. Intraparenchymal ICP monitoring consists of a thin cable with an electronic or fiberoptic transducer inserted directly into brain parenchyma. This technique can provide continuous measurements and can be implemented using a multi-lumen bolt to correlate with changes in other monitors, but is unable to drain CSF and has potential to lose accuracy (i.e., drift) over several days. The EVD and intraparenchymal ICP probe can be used in conjunction to achieve effective diagnostic and treatment utility.

Due to changing physiologic values and different pressure-volume relationships as children age, criteria for intracranial hypertension (ICH) are nebulous (4). Fifteen studies involving 857 pediatric patients have demonstrated association between ICH and poor outcome (4–19). One retrospective study found that use of ICP monitoring vs. no ICP monitoring was associated with reduced mortality in severely head injured patients (20). Another study utilized retrospective analysis to investigate 36 institutions using the Pediatric Health Information System database and found that hospitals with higher standardized ICP monitoring rates had better patient outcomes with lower rates of mortality or severe disability (21). A subsequent study by the same investigators used propensity-weighted effective analysis linking two national databases and reported no significant difference in functional survival between patients who underwent ICP monitoring and those who did not undergo ICP monitoring, but rather an association with monitoring and higher mortality, discharge to hospice, or either tracheostomy or gastrostomy tube placement (22). The authors cautioned that findings could be due to unmeasured differences between the groups. Current guidelines in pediatric TBI support a treatment threshold of 20 mm Hg (19), although there is uncertainty about how similar the autoregulatory curve changes with age (23).

### Brain Tissue Oxygenation

The brain has a high metabolic demand and contains limited stores of high-energy substrates. Continuous oxygen inflow is thus necessary to meet such demand. In TBI, fluctuations in oxygen delivery can emerge due to various processes and interventions. Techniques exist to provide continuous measurements of cerebral oxygenation.

Near-infrared spectroscopy (NIRS) provides non-invasive monitoring of cellular tissue oxygenation. Infrared light is emitted by diodes and detected by silicon phosphodiode optodes placed over the scalp of the frontal lobes. It is absorbed at wavelengths with the presence of oxygenated and deoxygenated hemoglobin whose concentration indexes cerebral oxygenation. The non-invasive nature of NIRS is advantageous, though it can be difficult to interpret in the setting of a scalp hematoma, intracranial hemorrhage, cerebral edema, and fluctuations in intracranial blood volumes (24, 25). In pediatric TBI, changes in NIRS values are associated with changes in ICP, mean arterial pressure and carbon dioxide content (26).

Continuous monitoring of cerebral oxygen partial pressure and temperature in brain tissue (P_bt_O_2_) can be measured using a micro-Clark electrode (27). This technique uses a closed electrochemical polarographic micro-cell for oxygen measurements and a thermocouple (type K) for temperature measurements. Four studies investigated the association of P_bt_O_2_ with clinical outcomes after pediatric TBI. One study analyzed over 8,000 h of monitoring from 46 children with severe TBI and observed that P_bt_O_2_ levels of 30 mmHg represented the highest combined sensitivity and specificity for favorable outcomes (28). The sensitivity was low (20%) and observations of elevated P_bt_O_2_ values in some patients with ICH and compromised CPP suggested the need to better understand the relationship of P_bt_O_2_ with outcomes. A prospective observational study of 52 children with severe TBI investigating the association of P_bt_O_2_ values and outcomes and found that worsened outcomes were associated with P_bt_O_2_ values <10 mmHg such associations were stronger with P_bt_O_2_ levels <5 mmHg persisting >1 h (29). A sub analysis of 28 children in this group revealed that in patients whose P_bt_O_2_ changed more in response to changes in PaO2 had worse outcomes (30). Current TBI guidelines recommend maintaining P_bt_O_2_ values >15 mm Hg in adults (31) and 10 mmHg in children (19). The recent BOOST-II trial was a randomized multicenter prospective clinical trial of 119 adult patients with severe TBI who were randomized to treatment protocols based on either ICP plus P_bt_O_2_ monitoring vs. ICP monitoring alone (32).The study revealed that a management protocol based on P_bt_O_2_ plus ICP monitoring reduced the proportion of time with brain tissue hypoxia. Results also showed that treatment informed on P_bt_O_2_ plus ICP monitoring was consistent with reduced monitoring and increased proportions of patients with good recovery as compared to ICP monitoring alone, although the study was not powered for clinical efficacy. The upcoming trial, BOOST-III will test the primary hypothesis that such dual monitoring therapy is associated with functional outcome after adult TBI. The Approaches and Decisions in Acute Pediatric TBI trial (ADAPT) has recently completed enrollment and will implement a comparative effectiveness strategy on multicenter data to investigate the association of P_bt_O_2_ monitoring with outcomes after pediatric TBI (33).

### Cerebral Blood Flow

Adequate P_bt_O_2_ and substrate delivery ultimately depends upon CBF. Several neurocritical care interventions are aimed at optimizing CPP, thus CBF monitoring techniques can help in guiding therapy. In children, CBF values increase from their lowest values at birth to peak at ages 3–5 years, then decrease toward adult levels (34, 35). It therefore remains critical for age-specific ranges to be considered and CBF values to be interpreted in context of metabolic demand.

Transcranial Doppler (TCD) ultrasonography is a non-invasive technique which provides continuous or intermittent bedside assessments of CBF. The technique utilizes the Doppler shift principle to derive red blood cell mean flow velocities (MFV) from pulsed ultrasound waves directed toward basal cerebral arteries, from which CBF can be inferred. TCD has traditionally been utilized for the assessment of cerebrovascular vasospasm (CVS) and arterial occlusions but has also shown utility in the assessment of several other physiologic processes including hyperemia, autoregulation, and critical closing pressure. In critically ill children, MFV are known to change with age (36). One study prospectively investigated 69 children with severe TBI who underwent TCD testing and observed that children with good outcomes were more likely to have normal than abnormal flow velocities, and no patient with a single low flow velocity measurement had good neurologic outcome (37). TCD was also used by the same investigators in a prospective observational study to observe the incidence of cerebral vasospasms after pediatric TBI, and they found that middle cerebral artery vasospasms occurred in 8.5% of children with moderate TBI and 33.5% of children with severe TBI. Basilar artery vasospasms occurred in 3% of children with moderate TBI and 21% of children with severe TBI (38). A recent survey of practices in pediatric neurocritical care centers showed that 74% of centers used TCD for clinical care, often for determining timing of neuroimaging, manipulating CPP, and deciding whether to perform surgical interventions (39).

Laser Doppler (LD) flowmetry functions similarly to TCD, particularly in assessing microcirculatory changes. A fiberoptic laser probe is placed onto brain parenchyma and detects light reflected by red blood cells to derive MFV. Changes in LD flowmetry are associated with impaired autoregulation (40), though the technique is prone to artifact produced by probe migration (41). Thermal diffusion (TD) flowmetry is an invasive technique that provides continuous quantitative brain perfusion measurements. This technique uses two thermistors within the probe, a proximal source set at the temperature of surrounding tissue and a distal censor heated 2 degrees Celsius higher. TD flowmetry takes advantage of the capacity of blood to dissipate heat to quantify CBF in units of mL/100 g/min (42). Current literature also indicates that TD values below 15 mL/100 g/min are associated with CVS in adult patients after aneurysmal subarachnoid hemorrhage (43). While these techniques are utilized in children with TBI in select centers (44), there is a lack of literature describing their findings.

### Cerebral Metabolism

Cerebral metabolism monitoring allows clinicians to better understand the effect of physiologic processes on the critically ill brain. Cerebral microdialysis (CMD) allows for direct measurement of cerebral metabolites including lactate, pyruvate, glycerol, glutamate, or glucose. The technique utilizes a catheter with a semipermeable dialysis membrane at its tip placed into brain parenchyma. Perfusate, an osmolar solution with electrolytic properties like CSF, passes along the membrane before exiting through outlet tubing. Microvials are then removed and placed in a bedside analyzer. In adult TBI, anaerobic metabolite patterns manifesting with elevated lactate or lactate-pyruvate ratios are associated with poor outcome (45–48).

A series investigating nine children with severe TBI found that a low glutamine/glutamate ratio was associated with increased morbidity, while a high ratio was associated with clinical improvement (49). Brain metabolism changes with increasing age, as myelination and synapse growth increase CBF during the first decade of life (50, 51). These phenomena likely play a role in what CMD parameters may be critical within the pediatric population, requiring future work to establish its effective use.

### Electroencephalography

Electroencephalography (EEG) is a technique that assesses cerebral cortical function through brain wave recordings. Historically, use of continuous EEG (cEEG) has been most fruitful in pediatric neurocritical care for detection of seizures (52). Retrospective work on critically ill children who underwent cEEG monitoring has shown that seizures independently contribute toward short-term outcomes (53). cEEG also has utility in monitoring depth of sedation, degree of encephalopathy (54), and trending cerebral ischemia (55). Intracortical EEG (iEEG) has been increasingly utilized in adult neurocritical care (56) and is associated with improved signal-noise ratio, detection of seizures not captured on cEEG, clarification of equivalent cEEG patterns and detection of cortical spreading depolarizations (57). One pediatric TBI study utilized iEEG showing that 3/11 patients had epileptiform abnormalities not captured on scalp EEG (58). Quantitative EEG (qEEG) utilizes mathematical algorithms to compress raw cEEG into graphical data. This can manifest as a color dense spectral array (CDSA) or rhythmic spectrogram, making detection of pathologic patterns such as seizures and ischemia easier to recognize for bedside clinicians. In the previously described study of pediatric TBI patients undergoing iEEG, both surface and intracortical alpha-delta ratios, a qEEG measure, were associated with CPP (58).

### Pupillometry

The pupilometer is a non-invasive handheld device that provides objective measures of pupil reactivity before and after a light stimulus. In management of ICP, abnormalities of pupil reactivity are often associated with neurological deterioration and poor outcome (59). Abnormal pupillary reactivity correlates with cerebral herniation, third nerve compression and brainstem perfusion (60). Traditional clinical methods of monitoring pupillary size and reactivity through observation with light are highly subjective (61), leading to the need for more sensitive and objective methods of detecting changes. In addition to providing objective measurements of pupil size, other variables including pupil reactivity (NPi), latency time and constriction and latency velocities can be assessed with pupillometry (62). Characterization of changes within these variables may help distinguish pathologic ICP increases in selected patients. A prospective study of 90 children without intracranial or ophthalmologic pathology was performed capturing pupillometry data, providing initial pediatric normative values (61).

## Approaches to Multimodal Monitoring in Pediatric Neurocritical Care

Multimodal monitoring (MMM) has allowed for a wealth of physiological data to be acquired in critically ill patients, but the interpretation of such complex, high-resolution data remains challenging. Integrated MMM platforms exist that output these data in time-synchronized formats to allow clinicians to explore real-time physiologic-interrelationships. One can explore trends in continuous quantitative values for physiologic variables such as ICP or P_bt_O_2_, as well as analysis of time-synchronized waveforms in ICP, TCD ultrasound MFV or EEG.

Various approaches can guide clinical management based on MMM data. Traditional methods prior to the advent of MMM focus on threshold-based algorithms. Under this approach, specific normative values are set for each physiologic variable explored and algorithms are designed to manage against specific perturbations. This methodology largely lacks exploration of the etiology of such perturbations and thus is limited in providing physiology-directed care. For instance, one may be able to treat against an ICP >20 mmHg under such an algorithm but may not consider whether such ICP elevation is related to hyperemia, a plateau wave, cerebral edema, or seizures.

Decision-making algorithms can be used to explore MMM data. Perturbations in specific physiologic variables can be initially identified, and trends in other variables may be used to better understand perturbations to direct care. For instance, if an ICP elevation is associated with an increase in P_bt_O_2_ and MFV, then this might suggest hyperemia and one may consider hyperventilation to reduce ICP by decreasing intracranial arterial blood volume. In contrast, if an ICP elevation is associated with a decrease in P_bt_O_2_ and MFV, this may suggest evolving cerebral edema and hypertonic saline could be considered.

Machine learning represents a powerful set of techniques in which multivariate data can be analyzed to discern inherent patterns. Cluster analysis has had wide use in genomics as a method to link individuals with shared characteristics to hypothesized groups. Using this technique to assess CMD in TBI patients, perturbed local chemistry was found to correlate with changes in CPP and ICP (63). Hierarchical cluster analysis has been utilized in TBI patients to distinguish specific physiologic states that were enriched for those who died, contracted an infection, and suffered multiple organ failure (64). A challenge to developing effective machine learning tools is the limited amount of clean data available and lack of annotation possible in a critical care environment.

An essential element to interpreting neurophysiologic data is determining whether one physiologic process mediates changes observed in other processes. Causal mediation analysis tests for such by linking treatment, outcome, and mediator variables. In a prospective studying observing the association of non-convulsive seizures with outcome in subarachnoid hemorrhage patients, mediated analysis linked clinical symptoms and serum biomarkers of inflammation as causal factors toward patient outcome (65).

**Figure 1:**
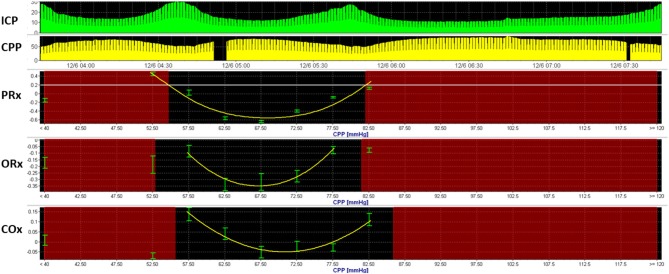
Optimal cerebral perfusion pressure based upon model-based indices of cerebral autoregulation. Multimodal monitoring data is recorded from a 3-year-old boy with severe TBI. Here, an optimal cerebral perfusion pressure (CPPopt) is estimated using three model-based indices of cerebral autoregulation, including the pressure reactivity index (PRx), oxygen-reactivity index (ORx), and cerebral oximetry index. U-shaped curves can be observed using all three indices, with CPPOpt ranging from 66 to 71 mmHg.

Model-based derivatives have emerged that combine physiologic variables into mathematical models to predict physiologic states. The pressure-reactivity index (PRx) (66), mean velocity index (Mx) (67), oxygen reactivity index (ORx) (68), and cerebral oxygenation index (COx) (69) are among several indices that explore changes in ICP, MFV, arterial blood pressure (ABP), P_bt_O_2_, and NIRS oxygen saturation to assess cerebral autoregulation. These indices utilize linear and multivariate regression analyses to formulate correlation coefficients within a moving window. When such derivations are explored over a range of vital signs, optimal conditions may be discernible (Figure 1). For instance, when PRx is observed over a range of CPP, an optimal CPP can be formulated, representing the lower threshold at which CPP may be maintained for an optimal autoregulatory state (70).

## Challenges and Future Directions

Adoption of emerging MMM techniques has led to an explosion of physiologic data. Like all data however, these can be subject to artifact, technical error, and misinterpretation. Challenges exist in effectively diagnosing physiologic states based on MMM and guiding care. Normative values must be recognized for all physiologic variables across ages and demographics. Artifact must be recognized and excluded. Invasive monitoring techniques increase the risk of infection and bleeding and these must be weighed in the decision of device placement. A study investigating the implementation of intracranial MMM in adult neurocritical care found that in 61 patients, 5% of patients experienced intracranial hematoma and 3% experienced a CNS infection (71). There are no studies that have investigated the association of MMM on outcomes or adverse events in pediatric TBI, and such work that includes age stratification is needed either through controlled-observational trials or comparative effectiveness studies. Ultimately, safe and effective methods of understanding brain physiology in real time and in a multimodal approach has the potential to prevent secondary brain injury in children with TBI and improve outcomes.

## Author Contributions

BA, BB, SF, and PA contributed toward the preparation, development, and critical review of this manuscript.

### Conflict of Interest

The authors declare that the research was conducted in the absence of any commercial or financial relationships that could be construed as a potential conflict of interest.
